# *Coriandrum sativum* and *Lavandula angustifolia* Essential Oils: Chemical Composition and Activity on Central Nervous System

**DOI:** 10.3390/ijms17121999

**Published:** 2016-11-30

**Authors:** Lucia Caputo, Lucéia Fátima Souza, Susanna Alloisio, Laura Cornara, Vincenzo De Feo

**Affiliations:** 1 Department of Pharmacy, University of Salerno, Via Giovanni Paolo II, 132, 84084 Fisciano (Salerno), Italy; lcaputo@unisa.it (L.C.); luceia.souza@ufrgs.br (L.F.S.); 2 Department of Agronomy, University of Rio Grande do Sul (UFRGS), 91501-970 Porto Alegre, Brazil; 3 ETT Spa, via Sestri 37, 16154 Genova, Italy; susanna.alloisio@ettsolutions.com; 4 Dipartimento di Scienze della Terra, dell’Ambiente e della Vita, University of Genoa, Corso Europa 26, 16132 Genoa, Italy; cornara@dipteris.unige.it

**Keywords:** *Lavandula angustifolia*, *Coriandrum sativum*, essential oil, linalool, cytotoxicity, Adenylyl Cyclase (ADCY), Extracellular Signal-Regulated Kinase (ERK), Multi Electrode Array (MEA), Central Nervous System (CNS)

## Abstract

The aims of this study are to determine the chemical composition of *Lavandula angustifolia* Mill. and *Coriandrum sativum* L. essential oils, to evaluate their cytotoxic effects in SH-SY5Y human neuroblastoma cells, to investigate whether an alteration of adenylate cyclase 1 (ADCY1) and of extracellular signal-regulated kinase (ERK) expression can take part in the molecular mechanisms of the essential oils, and to study their possible neuronal electrophysiological effects. The essential oils were obtained by hydrodistillation, and studied by GC and GC-MS. In the oils from *L. angustifolia* and *C. sativum*, linalool was the main component (33.1% and 67.8%, respectively). SH-SY5Y cells were incubated with different concentrations of essential oils and of linalool. Cell viability and effects on ADCY1 and ERK expression were analyzed using 3-(4,5-dimethylthiazol-2-yl)-2,5-diphenyltetrazolium bromide MTT and Western blotting, respectively. Variation in cellular electrophysiology was studied in primary cultures of rat cortical neurons with a multi-electrode array (MEA)-based approach. The essential oils and linalool revealed different cytotoxic activities. Linalool inhibited ADCY1 and ERK expression. Neuronal networks subjected to *L. angustifolia* and *C. sativum* essential oils showed a concentration-dependent inhibition of spontaneous electrical activity.

## 1. Introduction

Essential oils are used in aromatherapy due the effects of their constituents in the treatment and prevention of certain diseases related to the Central Nervous System [1,2]. The available literature reports anticonvulsant, antinociceptive, antiviral, antioxidant and anticancer effects of essential oils, among others [3,4].

Moreover, in the last few years, essential oil components have been evaluated for their mechanism of action and also to obtain lead compounds active in CNS [4].

*Lavandula angustifolia* Mill. (lavender, Lamiaceae) and *Coriandrum sativum* L. (coriander, Apiaceae) are aromatic plants recommended in folk medicine for relief of convulsion, anxiety and insomnia and in treatment of several neurological disorders [2,5,6]. The *Lavandula* genus is distributed in all Mediterranean regions and consists of about 20 species of small evergreen shrubs with aromatic foliage and flowers. Linalool, camphor, terpinen-4-ol, linalyl acetate, β-ocymene and 1,8-cineole are reported to be the main components of lavender essential oil [7,8]. This essential oil possesses different biological activities and many studies have investigated the effects of its major constituent, linalool, on brain activity or specific receptor populations [9,10,11].

Coriander is an annual herb originating from the Mediterranean region and cultivated in different parts the world. All parts of the plant are edible, the plant and its fruits are used as a spice in different Countries. In folk medicine, the fruits of coriander are recommended for treatment of anxiety, insomnia or for relief of nervousness [5]. Studies have reported coriander and its essential oil for sedative-hypnotic, anti-anxiety and antioxidant activities [12,13]. Linalool, geranyl acetate, nerol and neral are the main components of *C. sativum* essential oils [14].

Different studies concern the effects of the monoterpene alcohol linalool on Central Nervous System and its cytotoxicity to cancer cells. This compound appears to interact with different receptors. In fact, l-[^3^H] glutamate binding decreases with increasing concentrations of linalool, and the inhibition is dose-dependent [11]. Moreover, linalool interacts with NMDA receptor complex [15,16] and shows an inhibitory effect on acetylcholine release and channel open time in the mouse neuromuscular junction [17]. The inhalation of linalool provokes relaxing effects, and the combination of linalool and linalool oxide may be a useful mean to counteract anxiety [15,18,19] Linalool inhibited adenylyl cyclase activity by affecting the cAMP cellular content. [10,20].

Neuronal activity induces changes in synaptic plasticity that have been shown to require both PKA and ERKs [21]. ERKs are important components of activity-dependent signalling cascades within neurons and the modulation of their activity may be required for both synaptic plasticity and learning and memory [22].

In this study, we characterized the chemical composition of *L. angustifolia* and *C. sativum* essential oils, evaluated the cytotoxicity of linalool and the EOs against SH-SY5Y cell line and studied effects on the central nervous system. In particular, we consider the possible influence of the essential oils and linalool in cellular electrophysiology, and their role in expression of ADCY1 and ERK.

## 2. Results

### 2.1. Essential Oil Yields and Composition

Hydrodistillation of the aerial parts of *L. angustifolia* and of fruits of *C. sativum* furnished pale yellow oils in 5% and 2.1% yield on a dry mass basis, respectively. Table 1 shows the chemical composition of the two essential oils in percent; compounds are listed according to their elution order on a HP-5MS column. Altogether, 77 compounds were identified, 59 for *L. angustifolia*, accounting for 97.3% of the total oil, and 38% for *C. sativum*, accounting for 99.3% of the total oil.

In the oil from *L. angustifolia* linalool (33.1%), linalyl acetate (10.4%), 1,8-cineole (8.0%) and borneol (4.5%) are the main components. In the oil from *C. sativum*, the main compounds are linalool (67.8%), α-pinene (5.0%) and camphor (5.0%). Other compounds, in a lesser amount are *p*-cymene (2.8%), γ-terpinene (2.7%) and limonene (2.6%).

### 2.2. Cytotoxicity of Linalool, L. angustifolia and C. sativum Essential Oils

Cytotoxicity of linalool, *L. angustifolia* and *C. sativum* essential oils was evaluated using an MTT assay performed on the human neuroblastoma cell line (SH-SY5Y). After 24 h of treatment, the essential oils and linalool revealed different cytotoxic activities, with IC_50_ values of 334.5, 591.8 and 663.2 µg/mL, respectively.

**Figure 1:**
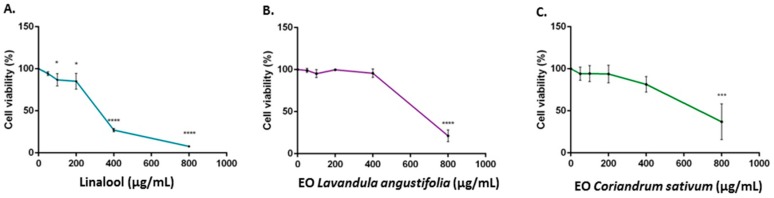
Cell viability calculated as a percentage after MTT assay. Cells were treated with different concentrations (50–800 µg/mL) of linalool (**A**); *L. officinalis* (**B**); and *C. sativum* (**C**) essential oils, for 24 h and solvent (DMSO, 0.1%) alone. Data are the mean ± SD of three experiments (* *p* < 0.05, *** *p* < 0.001, **** *p* < 0.0001 vs. DMSO).

Treatment of SH-SY5Y neuroblastoma cells with 800 µg/mL of linalool for 24 h resulted in a strong cytotoxic activity with 92% cell death. However, treatment with 800 µg/mL of *L. angustifolia* and *C. sativum* essential oils resulted in 78% and 63% cell death, respectively (Figure 1).

### 2.3. ADCY1, ERK: Western Blot Analysis

**Figure 2:**
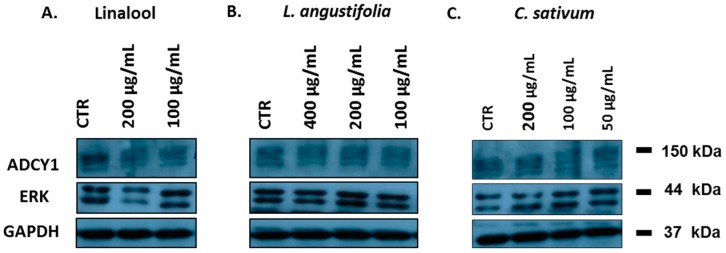
Representative Western blot of ADCY1 and ERK protein in SH-SY5Y treated with linalool (**A**); *L. angustifolia* essential oil (**B**); *C. sativum* essential oil (**C**).

**Figure 3:**
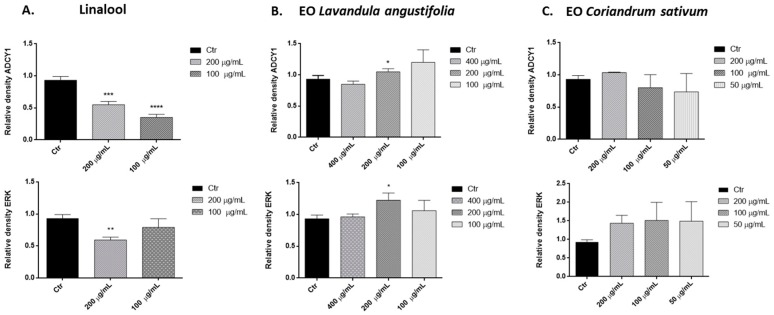
Relative expression levels of the ADCY1 and ERK protein in SH-SY5Y treated with linalool (**A**); *L. angustifolia* essential oil (**B**); *C. sativum* essential oil (**C**). Each panel shows densitometric analysis of bands in the control and treated groups. Values are the mean ± SD in each group (*n* = 3). * *p* < 0.05, ** *p* < 0.01, *** *p* < 0.001, **** *p* < 0.0001, compared to control (ANOVA followed by Dunnett’s multiple comparison test).

We investigated the effects of linalool, *L. angustifolia* and *C. sativum* essential oils in an SH-SY5Y cell line. More representative Western blots and quantitative densitometric analysis for ADCY1 and ERK protein expression in SH-SY5Y human neuroblastoma are shown in Figure 2 and Figure 3. Treatments of SH-SY5Y neuroblastoma cells with 200 and 100 µg/mL of linalool for 24 h significantly inhibited ADCY1 expression (Figure 3A). Treatment with 200 µg/mL of *L. angustifolia* appears to increase ADCY1 expression (Figure 3B). Linalool and *L. angustifolia* essential oil also have a similar effect on ERK protein expression. However, treatment with different concentrations of *C. sativum* essential oil had no significant effects on ADCY1 and ERK expression (Figure 3C).

### 2.4. Effects on Neuronal Activity

To evaluate if exposure to the selected essential oils affects neuronal spontaneous electrical activity, the mean firing rate (MFR) of primary cultures of rat cortical neurons was considered.

**Figure 4:**
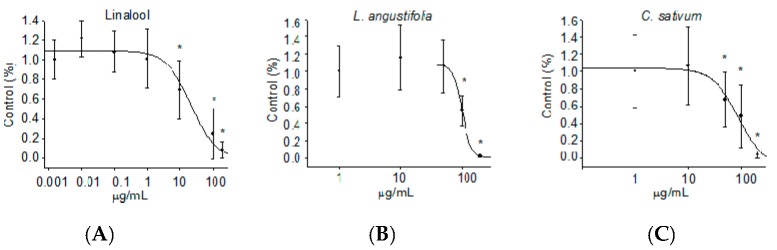
Effects of linalool (**A**); *L. angustifolia* (**B**); and *C. sativum* (**C**) essential oils on mean firing rate (MFR) of cortical cultures grown on microelectrode arrays. Each data point is the mean and SEM of 3 independent experiments (* *p* < 0.05 with respect to the normalised baseline values).

To evaluate the role of linalool, the major component of the two essential oils, in the reduction of neuronal networks functionality, we exposed neuronal cultures to increasing amounts of the compound. As illustrated in Figure 4A, linalool was considerably more potent than the two essential oils in reducing MFR, showing an IC_50_ of 25 µg/mL.

Neuronal networks subjected to *L. angustifolia* essential oil induced a concentration-dependent inhibition of activity with an IC_50_ value for MFR of 100 µg/mL and a total block at 200 µg/mL (Figure 4B). Differently, *C. sativum* essential oil reduced electrical activity with an IC_50_ value for MFR of 88 µg/mL, while a dose of 200 µg/mL completely blocked the firing activity (Figure 4C).

## 3. Discussion

Oxygenated monoterpenes are highly predominant in *L. angustifolia* and *C. sativum* essential oils, with a high percentage of linalool and a moderate concentration of limonene.

Linalool, linalyl acetate, 1,8-cineole, β-ocimene, terpinen-4-ol, and camphor are reported to be the main constituents of *L. angustifolia* essential oil [8]. However, the percentage of single constituents varies in different samples [23,24]. In this study, the major components of *L. angustifolia* oil were linalool (33.1%) and linalyl acetate (10.4%). This agrees with data by Koulivand and coworkers [8], who detected 51% linalyl acetate and 35% linalool. Borneol, (*E*)-β-ocimene, α-terpineol and (*Z*)-caryophyllene were found in moderate concentrations, comparable to the *L. angustifolia* essential oil from India [25].

The chemical composition of *C. sativum* essential oil may change depending on environmental conditions and is also affected by the duration and condition of storage [14]. In our sample, the amount of linalool was 67.3%, higher than in the essential oils analyzed by Khani and coworkers [26], and by Mandal and Mandal [5], who found percentages of linalool of 57.57% and 58%, respectively.

The main constituents in our *C. sativum* essential oil were linalool, α-pinene, camphor and geranyl acetate, in agreement with previous studies of Shahwar and coworkers [27] and Mandal and Mandal [5]. The observed variations in the relative percentages of single constituents may be attributed mainly to environmental conditions, method of harvesting, and methods used to obtain the essential oil [28,29,30].

Essential oils possess active components with in vitro cytotoxic activity towards various cancer cell lines. The cytotoxicity of essential oils is attributed to their lipophilic nature, causing damage to the plasma membrane. Their action can result in interference with cellular attachment, significant alteration in morphology, and eventually cell death [31].

The cytotoxic activity of linalool and of the two essential oils was evaluated in a human neuroblastoma cell line (SH-SY5Y) by means of an MTT assay.

Our results show that linalool, the main component of two essential oils, has a stronger cytotoxic activity against SH-SY5Y cells, with 92% cell death after a treatment with 800 µg/mL for 24 h. Ravizza and coworkers [32] demonstrated that linalool possesses antiproliferative effects against two human breast adenocarcinoma cell lines (MCF7 WT and MCF7 AdrR), and Sun and coworkers [33] reported similar results in human prostate cancer cells (DU145), at concentrations of 50 and 80 µM, respectively.

Comparing IC_50_ values, our findings indicated that *L. angustifolia* is more cytotoxic than *C. sativum* essential oil, probably due to components that act synergistically with linalool.

Prashar and coworkers [34] reported that *L. angustifolia* essential oil is cytotoxic to human skin cells in vitro (HMEC-1, HNDF, 153BR). Imelouane and coworkers [35] studied the cytotoxicity of the essential oils of *L. dentata* aerial parts and flower on five human cancer lines (P388D1, PC3, V79, U-373 MG, MCF7). They reported that the cytotoxicity of the flower oil is stronger than that of the oil from aerial parts. Conversely, to our knowledge no studies have been carried out to verify the cytotoxicity of *C. sativum* essential oil on neuroblastoma or other cell lines.

However, in our experiments the EC_50_ value for all the substances was >20 µg/mL, indicating that they were not cytotoxic as judged by the criterion set by the National Cancer Institute [36], which states that only natural substances with EC_50_ < 20 µg/mL are considered cytotoxic against the treated cells.

The available literature reports a well-established role for adenylyl cyclase in the regulation of multiple brain processes, such as synaptic plasticity, learning, and memory. Moreover, cross-talk between the cAMP signal transduction system and other signalling pathways, such as the ERK/MAP kinase regulatory system, has been described [37,38]. In this perspective, we carried out experiments to determine whether exposure to linalool and essential oils of *L. angustifolia* and *C. sativum* can affect these pathways in SH-SY5Y cells. Our results show that treatment with different concentrations of linalool decreases ADCY1 and ERK expression. Elisabetsky and coworkers [10,11], in a psychopharmacological in vivo evaluation of linalool, showed that this compound possesses dose-dependent sedative effects in the Central Nervous System. The inhibition of ADCY1 and ERK expression could explain the sedative effect described in other, previous studies [10,11].

Differently, the treatment with *L. angustifolia* essential oil showed an ADCY1 and ERK increased expression. We hypothesized that this effect probably is due to the presence of its derivative, linalyl-acetate (10.4%), which is not present in *C. sativum* essential oil.

Our results agree with those of Impey and coworkers [39] that showed that odorants generate transient increases in cAMP and Ca^2+^, both of which stimulate ERK activity in CNS neurons and PC12 cells.

*C. sativum* essential oil does not affect protein expression, despite its high content in linalool. In this oil linalyl acetate is absent, but possibly in the complex phytochemical composition of this essential oil there are one or more components that can counteract the linalool effect.

These findings suggest that an alteration of ADCY and ERK expression can be part of the molecular mechanisms underlying essential oil effects, but further studies are needed to confirm these findings.

In order to evaluate the potential effect of linalool and the essential oils of *L. angustifolia* and *C. sativum* on neuronal spontaneous electrical activity, we exposed rat neuronal networks grown on MEA to increasing concentrations of all products. The approach allowed, for the first time, an efficacy assessment of linalool and of the two essential oils. The results showed a concentration-dependent inhibition of neuronal networks firing activity for all the three agents, among which linalool is the most effective with an IC_50_ for MFR of 25 µg/mL. Conversely, the essential oils of *L. angustifolia* and *C. sativum* showed a lower efficacy as demonstrated by IC_50_ values of 100 and 88 µg/mL for *L. angustifolia* and *C. sativum*, respectively. Of note, the two essential oils revealed a similar efficacy, though the gas chromatography analysis demostrated a considerable difference in their content of linalool. In fact, considering that *L. angustifolia* essential oil contains 33.1% linalool, the IC_50_ value corresponding to the pure compound is about 33 µg/mL, which is near that obtained with linalool alone. It could therefore be assumed that the inhibiting effect of *L. angustifolia* essential oil is principally mediated by linaool.

Differently, *C. sativum* essential oil contains 67.3% of linalool and, consequently, the IC_50_ corresponding to the pure compound is about 59 µg/mL. This result could be explained by a reductive effect mediated by other components of *C. sativum* essential oil on the linalool-induced inhibition of electrical activity and shows that the combination of chemical mixtures leads to completely different effects from those obtained with each component applied singularly.

## 4. Materials and Methods

### 4.1. Plant Material

*Lavandula angustifolia* aerial parts and *Coriandrum sativum* fruits were collected in the Garden of Medicinal—Aromatic Plants in the Campus of the University of Salerno in July and August 2015, respectively. Plants were identified by Vincenzo De Feo. Voucher specimens (DF/367/2015 for *L. angustifolia* and DF/392/2015 for *C. sativum*) were stored in the Herbarium of the Pharmaceutical Botany Chair at the University of Salerno. Linalool was purchased by Sigma Italia, Milano.

### 4.2. Isolation of the Volatile Oil

One hundred grams of dried leaves of lavender and of dried fruits of coriander were ground in a Waring blender and then subjected to hydrodistillation for 3 h according to the standard procedure described in the European Pharmacopoeia [40]. The oils were solubilized in *n*-hexane, filtered over anhydrous sodium sulphate and stored under N_2_ at +4 °C in the dark until tested and analyzed.

### 4.3. GC-FID Analysis

Analytical gas chromatography was carried out on a Perkin-Elmer Sigma-115 gas chromatograph equipped with FID and data handling processor. The separation was achieved using a HP-5 MS fused-silica capillary column (30 m × 0.25 mm i.d., 0.25 μm film thickness). Column temperature: 40 °C, with 5 min initial hold, and then to 270 °C at 2 °C/min, 270 °C (20 min); injection mode splitless (1 μL of a 1:1000 *n*-hexane solution). Injector and detector temperatures were 250 °C and 290 °C, respectively. Analysis was also run by using a fused silica HP Innowax polyethylenglycol capillary column (50 m × 0.20 mm i.d., 0.25 μm film thickness). In both cases, helium was used as carrier gas (1.0 mL/min).

### 4.4. GC/MS Analysis

Analyses were performed on an Agilent 6850 Ser. II apparatus, fitted with a fused silica DB-5 capillary column (30 m × 0.25 mm i.d., 0.33 μm film thickness), coupled to an Agilent Mass Selective Detector MSD 5973; ionization energy voltage 70 eV; electron multiplier voltage energy 2000 V. Mass spectra were scanned in the range 40–500 amu, scan time 5 scans/s. Gas chromatographic conditions were as reported in the previous paragraph; transfer line temperature, 295 °C.

### 4.5. Identification of the Essential Oil Components

Most constituents were identified by gas chromatography by comparison of their Kovats retention indices (Ri) (determined relative to the t_R_ of *n*-alkanes (C_10_–C_35_)), with either those of the literature [41,42,43,44] and mass spectra on both columns with those of authentic compounds available in our laboratories by means NIST 02 and Wiley 275 libraries [45]. The components’ relative concentrations were obtained by peak area normalization. No response factors were calculated.

### 4.6. Human Neuroblastoma Cell Cultures

Human neuroblastoma (SH-SY5Y) cancer cells were cultured in in RPMI medium supplemented with 1% l-glutamine, 10% heat-inactivated fetal bovine serum (FBS), 1% penicillin/streptomycin (all from Sigma Aldrich, St. Louis, MO, USA) at 37 °C in an atmosphere of 95% O_2_ and 5% CO_2_.

### 4.7. Primary Neuron Cultures

Cortical neurons derived from enzymatically and mechanically dissociated cortex of day 18 embryonic Wistar SPF rat brain, as previously described [46]. After counting, 50,000–60,000 cells were plated on each poly-d-lysine (100 µg/mL) and Laminin (0.02 mg/mL) coated 60-electrode PEDOT-CNT MEA chip (Multi Channel Systems, Reutlingen, Germany). Neurons were maintained in neurobasal medium (NB) supplemented with 2% B27 and 1% Glutamax-I, and maintained in a humidified incubator at 37 °C in a 5% CO_2_ enriched atmosphere. Half volume of the medium was exchanged three times a week. As previously reported, experiments were carried out from 4 to 6 weeks in vitro, when neuronal networks are mature and both neuronal and glial cells are present [47]. Each preparation was tested in triplicate on neuronal networks derived from different isolations. All chemicals and reagents used for the preparation and maintenance of cultures were obtained from Invitrogen S.r.L. (Milan, Italy). All studies were performed according to the National Research Council’s guide for the care and use of laboratory animals by following protocols approved by the Institutional Animal Care and Use Committee.

### 4.8. MTT Bioassay

Human neuroblastoma cancer cells (SH-SY5Y) were plated (5 × 10^3^) in 96-well culture plates in 150 μL of culture medium and incubated at 37 °C in humidified 5% CO_2_. The day after, a 150 μL aliquot of serial dilutions of linalool (50–800 μg/mL) and *C. sativum* and *L. angustifolia* essential oils (50–800 μg/mL) were added to the cells and incubated for 24 h. DMSO alone was used as control. Cell viability was assessed through MTT (3-(4,5-dimethylthiazol-2-yl)-2,5-diphenyl tetrazolium bromide) assay. Briefly, 30 μL of MTT (5 mg/mL) was added and the cells incubated for additional 3 h. Thereafter, cells were lysed and the dark blue crystals solubilized with 30 μL of a solution containing 50%, *v*/*v*, *N*,*N*-dimethylformamide, 20%, *w*/*v*, SDS with an adjusted pH of 4.5. The optical density (OD) of each well was measured with a microplate spectrophotometer (Thermo Scientific Multiskan GO, Monza, Italy) equipped with a 520 nm filter. Cell viability in response to treatment was calculated as a percentage of control cells treated with DMSO at the final concentration 0.1% viable cells = (100 × OD treated cells)/OD control cells [48].

### 4.9. Extraction Proteins and Western Blotting

Cells were treated with different concentrations of linalool (100–200 μg/mL) and *L. angustifolia* (100–400 μg/mL) and *C. sativum* essential oils (50–200 μg/mL). The cells were collected after 24 h and lysed using the Laemmli buffer to extract total proteins. For Western Blot analysis, an aliquote of total protein was run on 8% SDS-PAGE gels and transferred to nitrocellulose. Nitrocellulose blots were blocked with 10% nonfat dry milk in Tris buffer saline 0.1% Tween-20 over night at 4 °C and incubated with primary anti-ADCY1 (Santa Cruz Biotechnology, Santa Cruz, CA, USA) for 3 h at room temperature. Immunoreactivity was detected through sequential incubation with horseradish peroxidase-conjugated secondary antibody (Amersham Biosciences, Pittsburgh, PA, USA) and enhanced chemiluminescence reagents (ImmunoCruz, Santa Cruz Biotechnology, Santa Cruz, CA, USA) [49].

### 4.10. Data Recordings, Signal Processing and Data Analysis

The spontaneous electrical activity was recorded by the USB MEA 120 INV 2 BC System from Multi Channel Systems (MCS GmbH, Reutlingen, Germany) as previously reported [46]. Briefly, the MEA chips were placed into the MEA Amplifier (Gain 1000×) and data were recorded by the MC_Rack software (MCS GmbH, Version 4.4.1.0) at a sampling rate of 10 kHz. A band pass digital filter (60–4000 Hz) was applied to the raw signal in order to remove electrical background noise. Only the electric signal overcomes the spike detection threshold of 5.5 times the standard deviation of the mean square root noise, was identified and recorded. Throughout the experiment, the cell cultures were maintained at 37 °C by a temperature controller (TC02, MCS GmbH) and in a controlled and humidified atmosphere (9% CO_2_, 19% O_2_ and 72% N_2_) to maintain the pH balance (pH was 7.0 ± 0.3). The data analysis was conducted by importing data (for MCS software, *.mcd files) into NeuroExplorer software (Littleton, MA, USA) and considering the network mean firing rate parameter (MFR; number of spikes/s). After exportation to Excel spreadsheets, data were averaged over several MEAs to create concentration curves for each treatment. The estimated IC_50_ values (half-maximal inhibitory concentration) were obtained by interpolating the normalised concentration–response curves of single treatments with the following four-parameter logistic function using SigmaPlot 8 software (Jandel Scientific, San Rafael, CA, USA):

f(*x*) = Max + (Min − Max)/(1 + (ε/*x*)β)

where the variable *x* is the concentration of the compound; the parameter Min is the minimum effect; the parameter Max is the maximum effect; the parameter ε is the concentration at which the effect is reduced by 50% (IC_50_); β is a parameter related to the maximum slope of the curve, which occurs at concentration ε.

### 4.11. Statistical Analysis

All experiments were carried out in triplicate. The data for each experiment were statistically analyzed using GraphPad Prism 6.0 software (GraphPad Software Inc., San Diego, CA, USA) followed by comparison of means (two-way ANOVA) using Dunnett’s multiple comparisons test, at a significance level of * *p* < 0.05. Mean ± SEM of replicate experiments (*n*) are indicated throughout.

## 5. Conclusions

This study complements previous works that characterized the psychopharmacological effects of linalool, *L. angustifolia* and *C. sativum* essential oils on the central nervous system. The analysis of essential oil composition reveals a higher linalool content in *C. sativum* than in *L. angustifolia* oil. Linalool exerts dose-dependent cytotoxic effects on the SH-SY5Y cell line and decreases the expression of ADCY1 and ERK, whereas treatment with *L. angustifolia* essential oil increases ADCY1 and ERK expression. In this study we also investigated the effects of two essential oils and their major constituent on the spontaneous electrical activity of rat neuronal cells. Our results reveal for the first time that linalool alone and the two essential oils affect, in a concentration-dependent manner, neural firing activity. Further studies are necessary to clarify the mechanism of action underlying the pharmacological effects in vivo.

## Figures and Tables

**Table 1:** Chemical composition of the essential oils of *L. angustifolia* (L) and *C. sativum* (C).

No.	Compound	L	C	Ki ^a^	Ki ^b^	Identification ^c^
1	α-Thujene	-	0.1	909	929	1,3
2	α-Pinene	0.8	5.0	922	939	1,2
3	Camphene	0.6	1.0	935	954	1,2
4	Thuja-2,4(10)-diene	0.1	-	957	960	1,2
5	Sabinene	-	0.7	961	975	1,2
6	β-Pinene	0.9	0.8	980	979	1,3
7	Myrcene	1.9	0.1	985	990	1,2
8	α-Phellandrene	0.2	-	991	1002	1,2
9	δ-3-Carene	0.9	0.1	1000	1008	1,2
10	α-Terpinene	0.2	-	1000	1017	1,3
11	*p*-Cymene	0.4	2.8	1009	1024	1,2
12	Limonene	2.1	2.6	1014	1029	1,2,3
13	1,8-Cineole	8.0	0.1	1017	1031	1,2
14	(*Z*)-β-Ocimene	0.6	1.1	1025	1037	1,2
15	(*E*)-β-Ocimene	1.4	0.1	1036	1050	1,2
16	γ-Terpinene	0.3	2.7	1046	1059	1,2
17	*cis*-sabinene Hydrate	0.2	t	1057	1070	1,2
18	*cis*-Linalool oxide	0.1	0.7	1063	1072	1,2,3
19	Terpinolene	0.7	0.5	1076	1088	1,2,3
20	Linalool	33.1	67.8	1099	1096	1,2,3
21	(2*E*)-Heptenyl acetate	0.2	-	1100	1113	1,2
22	Menth-en-1-ol	t	-	1109	1121	1,2,3
23	allo-Ocymene	1.3	-	1115	1132	1,2
24	*trans*-Pinocarveol	0.1	-	1125	1135	1,2
25	Camphor	11.0	5.0	1135	1146	1,2,3
26	Lavandulol	0.1	-	1153	1169	1,2
27	Borneol	4.5	0.3	1155	1160	1,2
28	Pinocarvone	0.1	-	1165	1164	1,2
29	neo-iso-Isopulegol	2.3	-	1166	1171	1,2
30	Terpinen-4-ol	-	0.3	1167	1177	1,2
31	*cis*-Linalool oxide	t	-	1172	1170	1,2,3
32	*p*-Cimen-8-ol	-	0.1	1176	1182	1,2
34	Menthol	0.2	-	1177	1171	1,2
35	α-Terpineol	1.6	0.6	1182	1188	1,2
36	Hexyl butanoate	0.5	-	1183	1192	1,2
37	Methyl Chavicol	-	0.1	1188	1196	1,2
38	Safranale	-	t	1197	1196	1,2
39	*n*-Decanal	-	t	1207	1201	1,2
40	Nerol	0.2	-	1215	1229	1,2
41	Citronellol	-	0.3	1217	1225	1,2
42	Hexyl-(2*E*)-butenoate	0.3	-	1228	1242	1,2
43	Neral	-	0.1	1230	1238	1,2
44	Linalyl acetate	10.4	-	1247	1257	1,2
45	Geraniol	-	2.0	1248	1252	1,2
46	Geranial	-	0.1	1268	1267	1,2
47	Iso-3-Thujanol acetate	0.1	-	1275	1270	1,2
48	Neo-3-Thujanol acetate	0.1	-	1281	1276	1,2
50	α-Terpinen-7-ale	0.1	-	1283	1285	1,2
51	Thymol	-	0.1	1296	1290	1,2
52	10-Undecenal	-	t	1294	1299	1,2
53	*p*-Cymen-7-ol	t	-	1297	1290	1,2
54	Terpinyl acetate	0.1	-	1315	1317	1,2
55	Mirtenyl acetate	t	0.2	1315	1326	1,2,3
56	Neryl acetate	-	t	1346	1361	1,2
57	α-Terpinyl acetate	0.8	-	1355	1349	1,2
58	(*E*)-2-undecenal	-	0.1	1359	1360	1,2
59	α-Cubebene	0.2	-	1368		1,2
60	α-Copaene	1.4	-	1374	1376	1,2
61	β-Cubebene	0.2	-	1378		1,2
62	Geranyl acetate	-	3.7	1382	1381	1,2
63	Longifolene	0.2	-	1394		1,2
64	(*Z*)-Caryophyllene	3.3	-	1408	1408	1,2
65	(*E*)-Caryophyllene	0.4	0.1	1413	1419	1,2,3
66	*cis*-Thujopsene	0.3	-	1424	1431	1,2
67	β-Copaene	0.3	-	1433	1432	1,2
68	α-Guaiene	0.3	-	1442		1,2
69	6,9-guaiadiene	0.9	-	1445	1444	1,2
70	allo-Aromadendrene	0.3	-	1458	1466	1,2
71	*cis*-Muurola-4(14,5)diene	0.2	-	1468	1468	1,2
72	β-Selinene	0.3	-	1486	1490	1,2
73	δ-Selinene	0.5	-	1490	1492	1,2
74	γ-Cadinene	0.2	-	1511	1513	1,2
75	Caryophyllene oxide	0.4	-	1572	1583	1,2,3
76	epi-α-Cadinol	0.2	-	1629	1640	1,2
77	β-Bisabolol	1.2	-	1672		1,2
	Total	97.3	99.3			
	Oxygenated Monoterpene	72.9	77.8			
	Monoterpene hydrocarbons	12.6	17.6			
	Sesquiterpene hydrocarbons	9.2	3.7			
	Oxygenated sesquiterpenes	1.6	-			

^a^ Kovats retention index on HP-5 MS column; ^b^ Kovats retention index on HP Innowax; ^c^ 1 = Kovats retention index, 2 = mass spectrum, 3 = coinjection with authentic compound; t = trace, less than 0.05 %; - absent.

## Abbreviations

ADCY1	Adenylyl Cyclase 1
CNS	Central Nervous System
ERK	Extracellular signal-Regulated Kinase
MEA	Multi Electrode Array
MTT	3-(4,5-dimethylthiazol-2-yl)-2,5-diphenyl tetrazolium bromide
OD	Optical Density
